# Vine-Inspired Twining Actuator: Cylindrical Hyper-Form-Closure Envelopment by Single Actuated Linkage

**DOI:** 10.3390/biomimetics11020125

**Published:** 2026-02-09

**Authors:** Jinnong Liao, Qihua Zhou, Yonglin Wang, Jinghua Chen, Yongsheng Luo, Gangfeng Liu, Meng Chen, Chongfeng Zhang, Jie Zhao

**Affiliations:** 1 State Key Laboratory of Robotics and System, Harbin Institute of Technology, Harbin 150001, China; 22b908038@stu.hit.edu.cn (J.L.); 25s108227@stu.hit.edu.cn (Y.W.); 23s008030@stu.hit.edu.cn (J.C.); 23b908055@stu.hit.edu.cn (Y.L.); jzhao@hit.edu.cn (J.Z.); 2 Shanghai Academy of Spaceflight Technology, Shanghai 201109, China; zhouqihuahit@126.com (Q.Z.); chenmeng@spacechina.com (M.C.); zhangchongfeng@spacechina.com (C.Z.)

**Keywords:** deployable mechanism, 1-DOF actuator, enveloping grasp, linkage mechanisms

## Abstract

Linkage mechanisms with fewer closed loops exhibit limited enveloping angles, whereas multi-loop designs increase complexity, compromise reliability, and introduce structural interference issues. This paper establishes the kinematic general formula of the N-layer Reverse Four-Bar Linkage, whose spiral enveloping mechanism is inspired by the twining growth of climbing plants. It reveals the variation law of the envelope angle with the closed-loop layer number *N*, and explores the influence of structural parameters on the configuration. It is found that when the symmetric length conditions of the two sets of opposing links are satisfied and the three-pair links meet the internal-angle constraint $\alpha_{1}=\alpha_{2}$, the mechanism exhibits self-similar topological characteristics, allowing the mechanism to maintain kinematic stability during multi-layer expansion. In terms of prototype implementation, the multi-link interference issues were successfully addressed by adopting slotted shaft-thrust bearing composite joints and a stepped arrangement design, leading to the development of an N=6 six-layer Reverse Four-Bar Linkage prototype. The prototype achieves a theoretical envelope angle of 450°, enabling hyper form closure grasping. It can stably grasp objects such as cylindrical objects with diameters ranging from 35 mm to 110 mm, effectively adapting to the grasping requirements of targets with various sizes and shapes. This provides a highly versatile and reliable grasping solution for industrial automation scenarios.

## 1. Introduction

**Figure 1:**
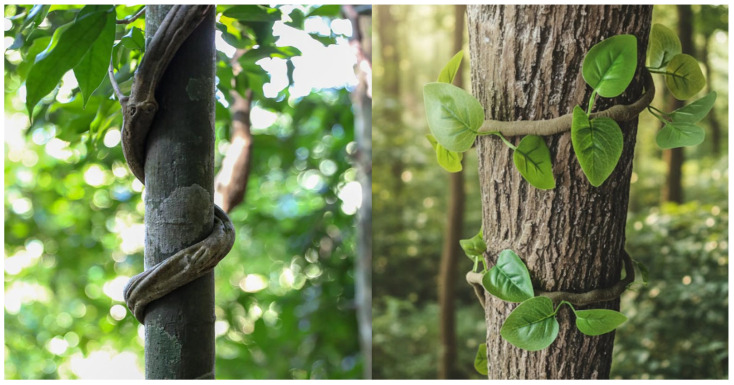
Trunk climbing and twining of vines.

In nature, many climbing plants (such as vines) achieve stable attachment and climbing by spirally twining around their supports (e.g., tree trunks), as illustrated in Figure 1. This efficient spirally envelopment strategy can adapt to cylindrical targets of different diameters via continuous contact points and provide robust grasping in complex environments. Inspired by this, we propose a bio-inspired twining mechanism that aims to translate the above spiral envelopment mechanism into an engineering solution. The mechanism adopts a one-DOF-driven multi-layer reverse four-bar linkage configuration, imitating the spiral twining motion of vines, thereby achieving hyper-form-closure grasping. This provides a novel approach for robust grasping of multi-scale targets.

The study of multi-loop linkages plays a crucial role in mechanical engineering and robotics. These systems are characterized by their ability to convert input motion into desired output motion through a series of interconnected links and joints. Recent advances demonstrate their growing sophistication: Pozhbelko [1] established a unified structure theory for open-, closed-, and mixed-loop mechanical systems, providing a universal framework for the topology, synthesis, and mobility analysis of multi-loop chains, which underpins the systematic design of complex mechanisms. Müller et al. [2] developed topological decomposition via kinematic model graphs, constructing hierarchical constraint descriptions using Lie group theory that can handle mechanisms with over 10 independent loops. For deployable systems, Cheng et al. [3] created umbrella-shaped mechanisms with 5:1 folding ratios through multi-layer linkage units, achieving 87% weight reduction compared to conventional designs. Tian et al. [4] designed a rolling robot with pure-translational nodes, reducing kinematic computation complexity by 60% through screw-based morphing strategies that enable 35° slope climbing. Liu et al. [5] proposed dual quaternion-based synthesis, achieving parametric design of 1-DOF multi-loop linkages with <0.05 arc-minute transmission errors between skew axes. Practical implementations include the symmetric four-bar carriage linkage developed by Kepler et al. [6], where symmetric geometry optimization reduces polygon effect-induced velocity fluctuations by 42%. Zhang et al. [7] developed a spatial closed-chain mechanism incorporating origami principles, achieving both an expansive workspace and high compaction ratio. Compared to open-chain mechanisms, closed-loop mechanisms exhibit superior stiffness and precision [8,9], which motivated the selection of this topology for our design.

The four-bar linkage, as the simplest closed-loop mechanism comprising four interconnected links with four joints, demonstrates remarkable versatility in motion generation and engineering applications. Its fundamental configuration consists of a fixed link, two moving links, and a coupler link transmitting complex motion patterns. This inherent adaptability enables widespread utilization across domains ranging from biomedical devices [10,11] to automotive systems [12,13]. To address the challenges of steering linkage layout in underslung suspension vehicles, Pichandi et al. [14] optimized the hardpoint placement of a four-bar linkage steering system through mathematical modeling. This approach ensures low Ackermann error and reduced bump steer, while achieving better handling and tire life compared to traditional Y-link solutions. Five-bar linkages have demonstrated significant versatility across multiple engineering domains, highlighted by several key developments. Their application extends to biomechanical systems, such as in gait rehabilitation devices, where they enable precise trajectory control for therapeutic purposes [15]. In robotics, research has focused on the optimal synthesis of these linkages, aiming to develop high-performance, singularity-free configurations suitable for precision manipulators [16]. Path synthesis and control strategies have been advanced using methods like shape invariants to optimize mechanism performance [17]. Furthermore, these linkages have been successfully implemented in the design of innovative, low-cost robotic systems, showcasing their practicality and accessibility for broader engineering applications [18]. These complexities limit scalability compared to four-bar linkages, whose one-DOF simplicity ensures robustness in high-volume applications like engines, relegating five-bar systems to specialized applications despite their adaptability.

Linkage mechanisms demonstrate unique advantages in cylindrical enveloping grasping, with their rigid transmission characteristics ensuring gripping stability. Meng et al. [19] proposed a linkage-driven underactuated hand capable of both precise pinching and powerful grasping through an optimized multi-linkage mechanism. Abdul Wahit et al. [20] further developed a 3D-printed prosthetic hand with four-bar linkages, demonstrating higher motion accuracy than tendon-driven systems in cylindrical grasping. Unde et al. [21] proposed a single-actuated multi-loop gripper that extends the grasping range to 30–145 mm diameter, but experimental results revealed mechanical efficiency degradation in multi-stage transmission. The wrapping-closure grasping forms a self-locking loop structure (with enveloping angles exceeding 360°), demonstrating superior anti-slip capability and dynamic stability compared to two-finger gripping, making it particularly suitable for reliable grasping of cylindroid workpieces (e.g., shafts and rollers) and flexible cables (e.g., wiring harnesses and hydraulic hoses).

While certain double-closed-loop linkage fingers provide strong gripping force, their enveloping angle is limited to approximately 213° and their adaptable diameter range is narrow [22]; pneumatic soft spiral grippers can achieve an enveloping angle exceeding 540°, but often come with structural complexity and moderate grip force [23]; origami-inspired structures can realize wrapping beyond 360° [24], yet suffer from insufficient grip force; and multi-closed-loop cable-driven grippers, although capable of reaching an enveloping angle of 517.6° [25], require coordinated control of four actuation units, resulting in system complexity. Therefore, developing novel mechanisms that simultaneously offer a large enveloping angle, high stability, and low complexity is particularly important. A more comprehensive quantitative comparative analysis is provided in Table 1 in Section 5.1 of this paper.

**Figure 2:**
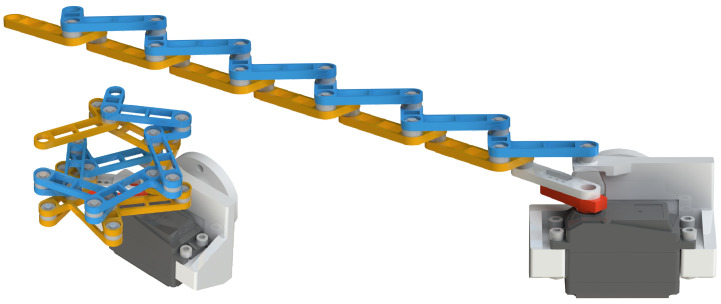
CAD model of one DOF N-layer reverse four-bar linkage (N = 6).

To address the dual challenges of limited enveloping angles in low-loop-number linkage and reliability degradation in multi-loop configurations, this study proposes an N-layer reversed four-bar linkage constructed through progressive superposition of three-pair links. The research conducts the kinematic modeling, parametric analysis, and functional prototyping to achieve two objectives: (1) developing a generalized formulation that quantifies the enveloping angle versus loop-layer relationship while identifying critical parameter influences, and (2) engineering a compact multi-loop mechanism with interference prevention for ultra-wide-angle grasping. Theoretical analysis employs homogeneous transformation for multi-closed-loop kinematics derivation, coupled with controlled-variable parameter studies, while engineering implementation features novel composite joints and stepped arrangements validated through physical prototype tests. The main contributions of this article can be summarized as follows:

(1) **Kinematic modeling and parameter analysis**: The kinematic formulation for N-layer reverse four-bar linkages was established, revealing the relationship between enveloping angle and number of layers. The influence of parameters on the N-layer reverse mechanism was analyzed. When two pairs of links are equal and $\alpha_{1}=\alpha_{2}$, the mechanism exhibits self-similar topological characteristics, maintaining kinematic stability during multi-layer expansion.

(2) **Prototype design and functional verification**: Using slotted shaft-thrust bearing composite joints and stepped arrangement, the multi-link interference problem was solved. An N-layer reverse four-bar linkage prototype (N = 6) was designed (The model is presented in Figure 2) and manufactured, achieving an enveloping angle of 450°. Successful grasping of cylindrical objects (35–110 mm diameter), 42 mm diameter bent pipes and 35 mm diameter ropes verified its engineering feasibility.

The remainder of this paper is organized as follows. Section 2 derives the kinematic formulation for N-layer reverse four-bar linkage and analyzes parameter influence patterns. Section 3 elaborates on the stair-stepped layout design for prototype interference prevention and the implementation scheme of each component. Section 4 validates functionality through grasping experiments on cylinders, bent pipes, and ropes. Section 5 discusses the cable-driven N-Layer reverse four-bar linkage and the comparison with existing actuators. Lastly, Section 6 concludes this article with a brief appraisal and future recommendation.

## 2. Design and Analysis of N-Layer Reverse Four-Bar Linkage

### 2.1. Design Objectives and Selection of Kinematic Chain

Based on the theory of form closure, if the motion degrees of freedom of an object are fully constrained under frictionless contact points, the grasp satisfies form closure. Form closure is the most conservative constraint, representing a complete kinematic constraint [26].

In the process of enveloping objects with linkage mechanisms, the contacting links are those directly interacting with the object, where their number determines the envelopment performance. The envelope angle is defined as the rotational angle from the initial to the final contacting link, achieving form-closure when $\beta_{\mathrm{Wrap}}\geq 180^{\circ }$ [22].

When the mechanism’s enveloping angle satisfies $\beta_{\mathrm{Wrap}}\geq 360^{\circ }$, the system achieves hyper form closure. At this stage, the driving mechanism completes $n=\lfloor \beta_{\mathrm{Wrap}}/360^{\circ }\rfloor$ full wrapping cycles, forming multi-layer helical constraint loops. The contact normal vectors in each closure layer generate complementary constraint fields through phase offsets, mathematically expressed as:

(1) $$\overset{n}{\underset{m=1}{⋂}}\mathrm{cone}\left(\left\{n_{i}^{\left(m\right)}\right\}\right)=\left\{0\right\}$$

where $n_{i}^{\left(m\right)}$ represents the normal vector at the *i*-th contact point in the *m*-th closure layer (m=1,2,...,n). The $\mathrm{cone}(\left\{n_{i}^{\left(m\right)}\right\})$ denotes the convex cone generated by non-negative linear combinations of normal vectors in that layer, and ${⋂}_{m=1}^{n}$ represents the intersection operation across all layers. The equation’s right-hand side being the zero vector {0} indicates no common non-zero displacement direction exists, proving that all disturbances δx≠0 will be blocked by at least one constraint layer, achieving full-direction kinematic locking.

When $\beta_{\mathrm{Wrap}}\in \left[180^{\circ },360^{\circ }\right]$ with n=1, the single-layer form closure condition becomes:

(2) $$\mathrm{cone}\left(\left\{n_{i}^{\left(1\right)}\right\}\right)\cap \left(-\mathrm{cone}\left(\left\{n_{i}^{\left(1\right)}\right\}\right)\right)=\left\{0\right\}$$

where $\mathrm{cone}(\left\{n_{i}^{\left(1\right)}\right\})$ represents the forward constraint cone covering all blockable displacement directions, while $-\mathrm{cone}(\left\{n_{i}^{\left(1\right)}\right\})$ denotes its symmetric reflection cone indicating permitted motion directions. This equation requires the forward and reflection cones to intersect only at the origin, ensuring any non-zero displacement δx must be constrained by either forward or reverse directional blocking.

The core design objectives of this one degree of freedom (DOF) N-Layer reversed four-bar linkage are:

- To achieve controllable switching between fully extended linear configuration and curved configuration, enabling envelopment of multi-scale cylindrical objects.
- Through geometric evolution of nested closed loops, to realize 360° envelope angle $\beta_{\mathrm{Wrap}}$ in bent state, achieving cylindrical surface wrapping capability and enhanced form-closed envelopment.

**Figure 3:**
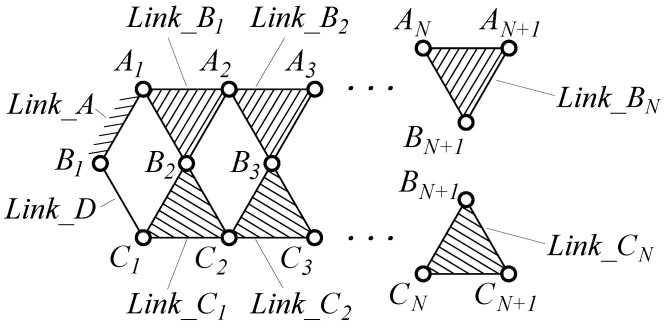
Kinematic chain of N-layer four-bar linkage.

As shown in Figure 3, the kinematic chain of the N-layer reverse four-bar linkage is a composite mechanism formed through topological extension of the Watt double-reverse type [22]. The primary configuration consists of a fixed frame (two-pair link) Link_A, a driving link (two-pair link) Link_D, and two three-pair links $\mathrm{Link}\_B_{1}$ and $\mathrm{Link}\_C_{1}$ forming the initial reverse four-bar loop. Subsequent layers (k=2,…,N) are constructed by adding pairs of three-pair links ($\mathrm{Link}\_B_{k}$ and $\mathrm{Link}\_C_{k}$) through shared revolute joints with preceding layers, ultimately creating *N* layer reverse four-bar closed loops. This hierarchical assembly results in a one DOF composite mechanism containing *N* reverse four-bar closed loops.

### 2.2. Kinematic Modeling

**Figure 4:**
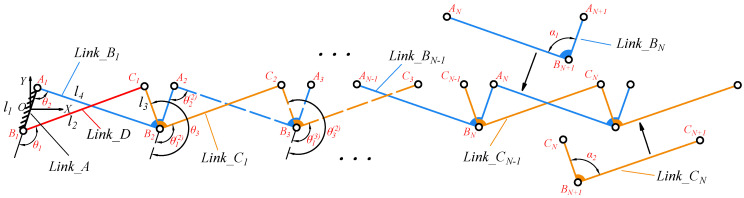
The overlapping process of one DOF N-layer reverse four-bar linkage.

The detailed assembly process of the N-layer reverse four-bar linkage is illustrated in Figure 4. For the four-bar reverse closed loop $A_{n}B_{n}C_{n}B_{n+1}$, $l_{1}$ is the length of $A_{n}B_{n}$, the coordinate origin of the fixed coordinate system is in the center of link $A_{1}B_{1}$. The angle between the positive direction of y-axis and vector $A_{1}B_{1}$ is frame deflection angle φ. $l_{2}$, $l_{3}$, and $l_{4}$ are the lengths of links $A_{n}B_{n+1}$, $B_{n+1}C_{n}$, and $B_{n}C_{n}$, respectively. The vector equation can be expressed as:

(3) $$A_{n}B_{n}+B_{n}C_{n}+C_{n}B_{n+1}+B_{n+1}A_{n}=0$$

Equation (3) can be written as two scalar equations

(4) $$\left[\begin{array}{ccc} \cos\theta_{2} & \cos\theta_{3} & -\cos\theta_{1}\\ \sin\theta_{2} & \sin\theta_{3} & -\sin\theta_{1} \end{array}\right]\left[\begin{array}{c} l_{2}\\ l_{3}\\ l_{4} \end{array}\right]=\left[\begin{array}{c} l_{1}\\ 0 \end{array}\right]$$

$\theta_{2}$ is the angle between link $A_{n}B_{n}$ and link $A_{n}B_{n+1}$, and $\theta_{3}$ is the angle between link $A_{n}B_{n}$ and link $B_{n+1}C_{n}$. $\theta_{2}$ and $\theta_{3}$ can be expressed as a function of the drive angle $\theta_{1}$ by solving the vector equation shown in Equation (3).

(5) $$\theta_{2}=2\arctan\left(\left(A_{1}+\sqrt{\left(A_{1}^{2}+B_{1}^{2}-C_{1}^{2}\right)}\right)/\left(B-C\right)\right)$$

where $A_{1}=-2l_{2}l_{4}s_{1}$, $B_{1}=-2l_{2}l_{4}c_{1}-2l_{1}l_{2}$, $C_{1}=l_{1}^{2}+l_{2}^{2}+l_{4}^{2}-l_{3}^{2}+2l_{1}l_{4}c_{1}$.

(6) $$\theta_{3}=2\arctan\left(\left(A_{2}-\sqrt{\left(A_{2}^{2}+B_{2}^{2}-C_{2}^{2}\right)}\right)/\left(B-C\right)\right)$$

where $A_{2}=-2l_{3}l_{4}s_{1},\;B_{2}=-2l_{3}l_{4}c_{1}-2l_{1}l_{3},\;C_{2}=l_{1}^{2}+l_{3}^{2}+l_{4}^{2}-l_{2}^{2}+2l_{1}l_{4}c_{1}$. The $s_{1}$ stands for $\sin\theta_{1}$ and $c_{1}$ stands for $\cos\theta_{1}$.

In three-pair links $Link\_B_{n}$ and $Link\_C_{n}$, $\alpha_{1}$ denotes the angle between $A_{n}B_{n+1}$ and $A_{n}B_{n}$ in $\mathrm{Link}\_B_{n}$, while $\alpha_{2}$ represents the angle between $C_{n}B_{n+1}$ and $B_{n}C_{n}$ in $\mathrm{Link}\_C_{n}$.

In the reverse four-bar closed loop, the remaining angles $\theta_{2}^{\left(k\right)}$ and $\theta_{3}^{\left(k\right)}$ can be analytically derived from the driving angle $\theta_{1}^{\left(k\right)}$ and link parameters using Equations (5) and (6). This allows recursive computation of joint angles for subsequent layers:

(7) $$\left\{\begin{array}{cc} \theta_{1}^{\left(k\right)}=\theta_{3}^{\left(k-1\right)}-\theta_{2}^{\left(k-1\right)}+\alpha_{1}-\alpha_{2}, & \mathrm{inter-layer}\;\mathrm{angle}\;\mathrm{transfer}\\ \theta_{2}^{\left(k\right)}=f_{\theta_{2}}\left(\theta_{1}^{\left(k\right)}\right), & \mathrm{defined}\;\mathrm{by}\;\mathrm{Equation}\;(5)\\ \theta_{3}^{\left(k\right)}=f_{\theta_{3}}\left(\theta_{1}^{\left(k\right)}\right), & \mathrm{defined}\;\mathrm{by}\;\mathrm{Equation}\;(6)\\ k=2,3,\ldots ,N, & \mathrm{layer}\;\mathrm{index} \end{array}\right.$$

With the recursive angle formulas derived above, the homogeneous transformation matrix (combining translation and rotation) can be employed to establish the pose mapping relationships between layers, enabling the computation of key point coordinates in the mechanism:

The translation transformation matrix describes pure translational motion in 3D space, represented in homogeneous coordinates as:

(8) $$T\left(d_{x},d_{y},d_{z}\right)=\left[\begin{array}{cccc} 1 & 0 & 0 & d_{x}\\ 0 & 1 & 0 & d_{y}\\ 0 & 0 & 1 & d_{z}\\ 0 & 0 & 0 & 1 \end{array}\right]$$

where $d_{x}$, $d_{y}$, and $d_{z}$ represent the displacement components along the X-axis, Y-axis, and Z-axis, respectively.

For planar linkage mechanisms (all motions confined to XY plane), only pure rotation about Z-axis is needed. The rotation matrix about the Z-axis by angle Φ is:

(9) $$R_{z}\left(\Phi \right)=\left[\begin{array}{cccc} \cos\Phi & -\sin\Phi & 0 & 0\\ \sin\Phi & \cos\Phi & 0 & 0\\ 0 & 0 & 1 & 0\\ 0 & 0 & 0 & 1 \end{array}\right]$$

where Φ is the rotation angle in right-handed coordinates (counterclockwise direction is positive).

The homogeneous transformation matrices $T_{1}$ through $T_{7}$ required for computing the pose matrices of all points in the first closed loop are defined as follows:

(10) $$\left\{\begin{array}{cc} T_{1} & =R_{z}\left(\phi \right)\\ T_{2} & =T(0,l_{1}/2,0)\\ T_{3} & =T(0,-l_{1}/2,0)\\ T_{4} & =R_{z}\left(\psi_{T_{4}}\right)\\ T_{5} & =T(l_{4},0,0)\\ T_{6} & =R_{z}\left(\psi_{T_{7}}\right)\\ T_{7} & =T(l_{2},0,0) \end{array}\right.$$

where $\psi_{T_{4}}=\theta_{1}-\pi /2$, $\psi_{T_{7}}=\theta_{2}-\pi /2$.

Similarly, the transformation matrices $C1T_{n}$ through $C6T_{n}$ (n≥2) required for computing pose matrices in subsequent closed loops are defined as:

(11) $$\left\{\begin{array}{c} C1T_{n}=R_{z}\left(\psi_{C1T_{n}}\right)\\ C2T_{n}=T\left(l_{4},0,0\right)\\ C3T_{n}=R_{z}\left(\psi_{C3T_{n}}\right)\\ C4T_{n}=T\left(l_{1},0,0\right)\\ C5T_{n}=R_{z}\left(\psi_{C5T_{n}}\right)\\ C6T_{n}=T\left(l_{2},0,0\right) \end{array}\right.$$

where $\psi_{C1T_{n}}=\theta_{1}^{\left(n\right)}-\theta_{2}^{\left(n-1\right)}$, $\psi_{C3T_{n}}=\pi -\alpha_{1}$, $\psi_{C5T_{n}}=\theta_{2}^{\left(n\right)}-\pi /2$.

The hierarchical pose relationships of multi-layer planar linkage mechanisms can be established through recursive chained multiplication of 4 × 4 homogeneous transformation matrices:

(12) $$\begin{array}{c} \left\{\begin{array}{cc} B_{n} & =\left\{\begin{array}{cc} T_{1}\cdot T_{2} & n=1\\ T_{1}\cdot T_{2}\cdot T_{6}\cdot T_{7} & n=2\\ A_{n-1}\cdot C_{5}T_{n-1}\cdot C_{6}T_{n-1} & n>2 \end{array}\right.\\ C_{n} & =\left\{\begin{array}{cc} T_{1}\cdot T_{3}\cdot T_{4}\cdot T_{5} & n=1\\ T_{1}\cdot T_{2}\cdot T_{6}\cdot T_{7}\cdot C_{1}T_{2}\cdot C_{2}T_{2} & n=2\\ B_{n}\cdot C_{1}T_{n}\cdot C_{2}T_{n} & n>2 \end{array}\right.\\ A_{n} & =\left\{\begin{array}{cc} T_{1}\cdot T_{3} & n=1\\ T_{1}\cdot T_{2}\cdot T_{6}\cdot T_{7}\cdot C_{3}T_{2}\cdot C_{4}T_{2} & n=2\\ B_{n-1}\cdot C_{5}T_{n-1}\cdot C_{6}T_{n-1} & n>2 \end{array}\right. \end{array}\right. \end{array}$$

This recursive system constructs the kinematic chain through hierarchical conditional expressions. The initial level (n=1) defines the fundamental motion transformation, the second level (n=2) introduces cross-coupling terms, while higher levels (n>2) achieve automatic parameter propagation through recursive relations. Each transformation matrix contains both rotational and translational components, whose combined operations accurately describe the composite motion characteristics of multi-link mechanisms.

The end-effector coordinates are derived directly from the terminal transformation matrix, representing a coordinate mapping in homogeneous coordinate space:

(13) $${\left[\begin{array}{cccc} x_{P} & y_{P} & z_{P} & 1 \end{array}\right]}^{⊤}=P\cdot {\left[\begin{array}{cccc} 0 & 0 & 0 & 1 \end{array}\right]}^{⊤}$$

The envelope angle of N-layer reverse four-Bar linkage is defined as the rotation angle from $B_{1}C_{1}$ to $B_{n+1}C_{n+1}$, the envelope angle $\beta_{\mathrm{Wrap}}$ is calculated by:

(14) $$\beta_{\mathrm{Wrap}}=\psi_{T_{4}}-\left(\psi_{T_{7}}+\sum_{k=2}^{n}\left(\psi_{C3T_{k}}+\psi_{C5T_{k}}\right)+\psi_{C1T_{n+1}}\right)$$

Under the specific condition where the internal angles $\alpha_{1}=\alpha_{2}$ in three-pair links $\mathrm{Link}\_B_{n}$ and $\mathrm{Link}\_C_{n}$, the calculation of the envelope angle $\beta_{\mathrm{Wrap}}$ demonstrates significant simplification. The geometric symmetry of the mechanism eliminates higher-order nonlinear coupling terms, reducing the envelope angle to a linear combination of the parameter $\theta_{2}$ and the three-pair link’s internal angle $\alpha_{1}$ (the envelope angle is solely related to the variable driving angle $\theta_{1}$, retaining only the indirect nonlinear dependence of $\theta_{2}$ on $\theta_{1}$):

(15) $$\beta_{\mathrm{Wrap}\_\mathrm{EqualAlpha}}=\left(1-n\right)\theta_{2}+\left(n-1\right)\alpha_{1}\;(\mathrm{where}\;\alpha_{1}=\alpha_{2})$$

### 2.3. Torque Transmission Efficiency

**Figure 5:**
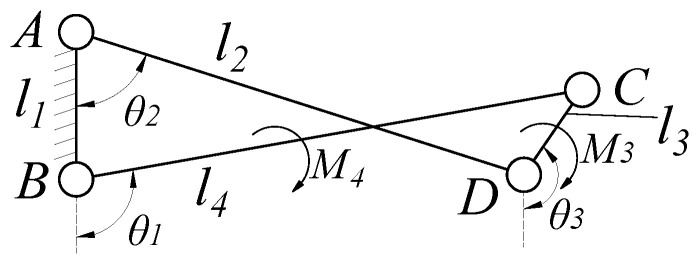
Schematic of a Single Reverse Four-Bar Closed Loop.

As the magnitude and direction of the contact forces during the grasping process are difficult to predict, the analysis of the mechanism’s force transmission characteristics primarily focuses on torque transmission. First, the force transmission efficiency of a single reverse four-bar mechanism is analyzed. The four-bar closed loop is shown in Figure 5. Assume the link length parameters, the driving angle $\theta_{1}$, and the magnitude of the driving torque $M_{4}$ acting on the driving link $l_{4}$ are known. It is also assumed that a torque $M_{3}$ acts on the hinge *C*, which connects the frame to link CD, to balance link CD. A static analysis is performed on each link. By solving the force and moment equilibrium equations shown in Equation (16), the relationship between the input torque $M_{4}$ and the output torque $M_{3}$ can be obtained, as shown in Equation (17).

(16) $$\left\{\begin{array}{c} \sum F_{x}=0\\ \sum F_{y}=0\\ \sum M=0 \end{array}\right.$$

(17) $$M_{3}=M_{4}\cdot \frac{l_{3}}{l_{4}}\cdot \frac{\sin(\theta_{2}-\theta_{3})}{\sin(\theta_{2}-\theta_{1})}$$

Therefore, the Torque Transmission Ratio (TTR) for a single closed loop is defined as the ratio of the output torque to the input torque:

(18) $$\mathrm{TTR}=\frac{M_{3}}{M_{4}}=\frac{l_{3}}{l_{4}}\cdot \frac{\sin(\theta_{2}-\theta_{3})}{\sin(\theta_{2}-\theta_{1})}$$

The *N*-layer mechanism proposed in this paper adopts a self-similar topological structure, meaning each closed loop shares identical link lengths and kinematic relationships. Consequently, under the same configuration, the TTR of each individual closed loop is equal, denoted as $\eta_{\mathrm{unit}}$. When *N* such closed-loop units are connected in series, the overall torque transmission efficiency of the system exhibits a multiplicative effect. Neglecting the minor influence of inter-layer coupling, the relationship between the final output torque $M_{3,N}$ at the *N*-th layer and the input torque $M_{4,1}$ at the first layer can be approximated as:

(19) $${\mathrm{TTR}}_{\mathrm{total}}=\frac{M_{3,N}}{M_{4,1}}\approx {\left(\eta_{\mathrm{unit}}\right)}^{N}\cdot {\left(\eta_{f}\right)}^{N}$$

where ${\left(\eta_{\mathrm{unit}}\right)}^{N}$ represents the cumulative multiplication of the ideal force transmission, and ${\left(\eta_{f}\right)}^{N}$ denotes the cumulative multiplication of joint friction losses ($\eta_{f}<1$). This formula clearly reveals that the overall force transmission efficiency decreases exponentially as the number of layers *N* increases. This is an inherent characteristic of multi-layer serial mechanisms, necessitating a design trade-off between the gain in enveloping angle and the attenuation of torque.

**Figure 6:**
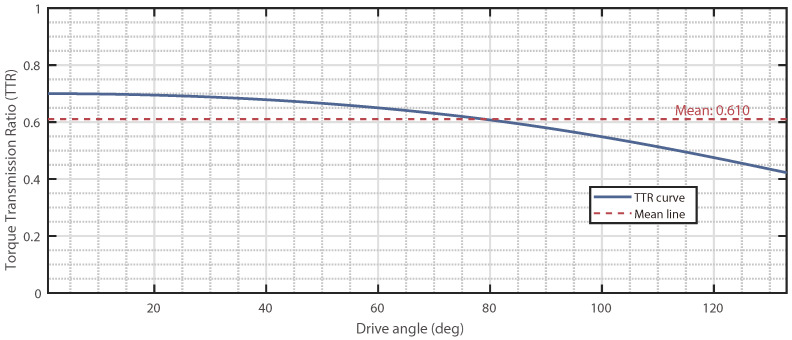
Torque transmission efficiency of a single reverse four-bar closed loop ($\alpha =90^{\circ }$, short links: 20 mm, long links: 50 mm).

Taking the prototype parameters from Section 3.2 of this paper as an example, the torque transmission efficiency of the first four-bar closed loop was calculated, and the result is shown in Figure 6.

As shown in the figure, the torque transmission efficiency increases as the drive angle decreases (corresponding to the mechanism’s motion from extension to bending), with an overall average efficiency of 0.61. This trend indicates that the mechanism possesses a relatively higher torque transmission capability when it reaches the final bent and wrapped configuration for grasping. For the N=6 layer serial mechanism studied in this paper, its overall torque transmission efficiency can theoretically be approximated as the product (cumulative multiplication) of the single-layer efficiencies. If estimated using the average efficiency of 0.61, the theoretical total efficiency is approximately $0.61^{6}\approx 0.05$. This implies that the output torque at the end effector will be significantly attenuated due to transmission through the layers. It is noteworthy that although the torque transmission efficiency decreases layer by layer, leading to a reduction in the effective output force at the end, the core grasping principle of this mechanism lies in form closure and even hyper form closure. Its stability does not rely on continuously applying a large gripping force at the end effector. Instead, it is achieved through the multi-link enveloping configuration of the mechanism, which forms a series of non-collinear, discrete contact constraints around the object. As long as these constraints can constitute force closure in the force space, stable grasping is achieved. Therefore, even if the output torque is attenuated after multi-layer transmission, as long as the mechanism can successfully complete the intended enveloping motion, the geometric constraints it forms are themselves sufficient to reliably grasp and hold the object.

### 2.4. Parameters Analysis

**Figure 7:**
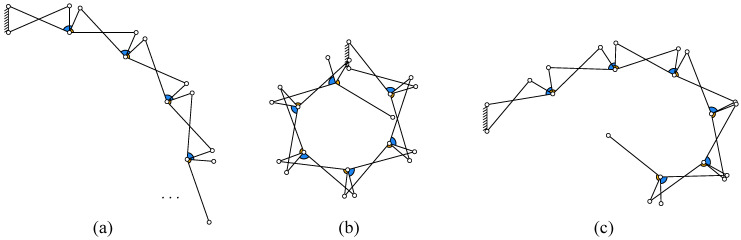
Envelope direction and inner envelope loop shapes under parameter variations. (**a**) Case with downward envelope direction. (**b**) Inner envelope loop under unsatisfied symmetric length conditions. (**c**) Inner envelope loop with unequal internal angles of three-pair links.

The enveloping characteristics of the N-layer reverse four-bar linkage are jointly determined by structural and kinematic parameters. The structural parameters include the frame deflection angle ϕ, the internal angles of two three-pair links $\alpha_{1}$ and $\alpha_{2}$, and four link lengths $l_{1}$, $l_{2}$, $l_{3}$, and $l_{4}$. The kinematic parameter is the primary input variable $\theta_{1}$, which controls the configuration transformation. The enveloping direction is defined as the trend orientation of subsequent closed loops when the frame and opposing link in the first reverse four-bar closed loop are parallel. As shown in Figure 7a, the enveloping direction in this diagram is downward.

Since the frame deflection angle ϕ only affects the installation position and has no impact on the mechanism, it will not be discussed here. The following analyzes the influence of other parameters on the enveloping characteristics of the N-layer reverse four-bar linkage:

**(1) Four link length parameters $l_{1}$, $l_{2}$, $l_{3}$, and $l_{4}$.** When maintaining the three-pair link angles $\alpha_{1}=\alpha_{2}$ with other parameters fixed, the four-bar lengths exhibit directional regulatory effects on the multi-layer closed-loop enveloping trend direction: increasing the lengths of $l_{1}$ or $l_{4}$ drives subsequent closed-loop trajectories to deviate outward from the baseline enveloping direction, while extending $l_{2}$ or $l_{3}$ enhances the contraction trend of the closed-loop system toward the core enveloping zone. When the symmetric length conditions $l_{1}=l_{3}$ and $l_{2}=l_{4}$ are violated, the inner envelope loop of the mechanism predominantly exhibits major segment-like morphology (the major segment being the planar region bounded by a chord and major arc). This non-ideal enveloping configuration leads to disordered contact force distribution, thereby degrading the enveloping effectiveness. The inner envelope loop under unsatisfied symmetric length conditions is shown in Figure 7b.

**(2) Internal angles $\alpha_{1}$ and $\alpha_{2}$ of two three-pair links.** When $\alpha_{1}\neq \alpha_{2}$ (with other structural parameters held constant and $l_{1}=l_{3}$, $l_{2}=l_{4}$), it becomes difficult to achieve near-linear configurations. The inner envelope loop predominantly exhibits major segment-like morphology. When $\alpha_{1}>\alpha_{2}$, subsequent closed loops deviate further from the enveloping trend direction, whereas when $\alpha_{1}<\alpha_{2}$, they converge closer to the enveloping trend direction. The inner envelope loop with unequal internal angles of three-pair links is depicted in Figure 7c.

When $\alpha_{1}=\alpha_{2}$, as shown in Equation (7), all closed loops exhibit identical angles ($\theta_{1}=\theta_{2}=\theta_{3}$ for each loop) with simplified general formulation. Link_B and Link_C having identical geometric parameters can be consolidated into a single standardized component. According to Grashof’s criterion, this four-bar closed-loop configuration becomes a double-crank mechanism capable of full rotation (operating as a reverse four-bar when the input angle θ ranges from $0^{\circ }$ to $180^{\circ }$, and as a forward four-bar from $180^{\circ }$ to $360^{\circ }$). This configuration yields simplified kinematic equations and enhanced manufacturing economy.

Under this condition, the mechanism achieves long-stroke wrapping capability through rigid closed-chain configuration while maintaining one-DOF actuation, enabled by its self-similar topological characteristics. This topologically self-similar structure maintains kinematic stability during multi-layer expansion while ensuring continuous satisfaction of form-closure conditions.

**Figure 8:**
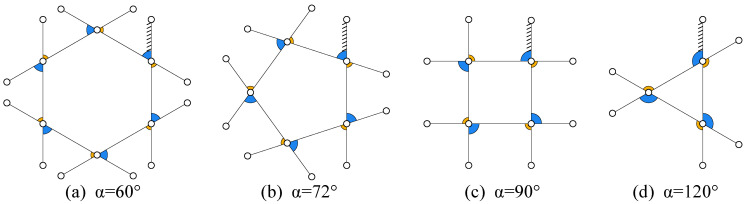
Bending limit configuration envelopes corresponding to different values of $\alpha_{1}$. (**a**) α = 60°. (**b**) α = 72°. (**c**) α = 90°. (**d**) α = 120°.

When $\alpha_{1}=\alpha_{2}$, $l_{1}=l_{3}$, and $l_{2}=l_{4}$, and when the ternary joint angle $\alpha_{1}$ takes specific values, the mechanism forms a closed regular *M*-gon in its bending limit configuration with driving angle $\theta_{1}=0$ (where all links become parallel) and $\theta_{2}=\theta_{3}=0$. The geometric shape is completely determined by $\alpha_{1}$, with the system’s internal angle sum satisfying $S_{\mathrm{extreme}}=M\left(\pi -\alpha_{1}\right)$. When the number of mechanism layers *n* exceeds *M* (n>M), the additional closed loops will progressively overlap. The theoretical internal angle sum for a regular *M*-gon is $S_{\mathrm{regular}}=\left(M-2\right)\pi$. When $\alpha_{1}$ exactly divides $360^{\circ }$ (i.e., $\alpha_{1}=360^{\circ }/M$ where M≥3 is an integer), the equality $S_{\mathrm{extreme}}=S_{\mathrm{regular}}$ holds, causing the bending limit configuration envelope to form a perfect regular *M*-gon whose number of sides is uniquely determined by $M=360^{\circ }/\alpha_{1}$. Typical examples include $\alpha_{1}=90^{\circ }$ yielding a quadrilateral (M=4) and $\alpha_{1}=120^{\circ }$ producing an equilateral triangle (M=3). For cases where $\alpha_{1}$ cannot divide $360^{\circ }$ exactly, the envelope becomes an irregular polygon with an effective side number $M_{\mathrm{eff}}=360^{\circ }/\alpha_{1}$ that is non-integer, leading to phase accumulation errors in the motion trajectory. The bending limit configuration envelopes corresponding to various $\alpha_{1}$ values are illustrated in Figure 8.

## 3. Prototype Implementation

### 3.1. Selection of Parameters

According to Section 2.3, when $\alpha_{1}=\alpha_{2}$ and $l_{1}=l_{3}$, $l_{2}=l_{4}$, the mechanism exhibits self-similar topological characteristics with uniform envelope curves. Under this condition, the mechanism parameters can be simplified to three variables: the long link length $L_{L}$, the short link length $L_{S}$, and the internal angle α of the ternary links.

The long link length $L_{L}$ is set to 50 mm based on the average proximal phalanx length of adult fingers (ranging 45–55 mm), which can satisfy daily grasping operations. The short link length $L_{S}$ is chosen as 20 mm, which not only meets the mechanical design requirement of having a distance greater than 15 mm between two rotational pairs, but also matches the size of adult metacarpophalangeal (MCP) joints.

The selection of internal angle α depends on the geometric characteristics of target objects. Although smaller α values can provide more envelope surfaces, they require a larger number of closed loops *N* to achieve hyper form closure. In this study, α is set to 90 degrees with N=6, achieving an envelope angle of 450 degrees in extreme bending configurations, which enables certain degree of wrapping capability (the maximum envelope angle of the mechanism in this state is $\left(N-1\right)\cdot \alpha_{1}$).

### 3.2. Prototype Construction

To validate the winding mechanism and performance of the described N-layer reverse four-bar linkage, and to explore the engineering implementation path from planar (2D) motion to spatial (3D) enveloping, this work designed and constructed a modular physical prototype. The core design concept of this prototype is “modular spatial stacking”: multiple closed-loop units (single-layer modules), which move independently and precisely within a 2D plane, are sequentially connected and arranged along the third spatial dimension perpendicular to that plane. This arrangement synthesizes a complete actuator capable of continuous helical enveloping in 3D space. The specific structural implementation is described as follows.

**Figure 9:**
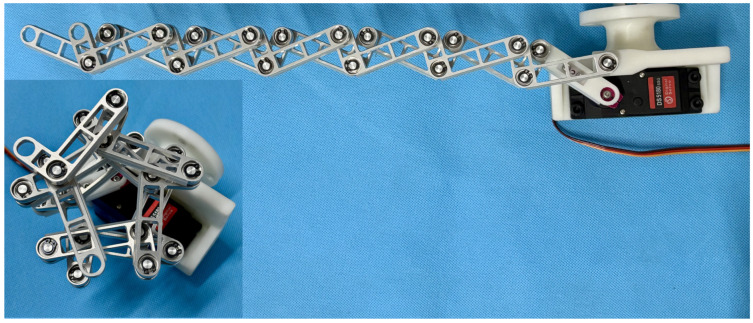
Physical prototype of one DOF N-layer reverse four-bar linkage (N = 6).

The main frame of the prototype is manufactured via 3D printing with integrated servo mounting positions, where the driving rod is rigidly connected to the servo arm through multiple bolts, enabling its attachment to a Franka Emika robotic arm. The standardized single link combines rods B and C into a unified component through symmetric design, utilizing aluminum alloy material with hollowed-out midsections for weight reduction. The revolute joint assembly employs a multi-layer structure consisting of retaining rings, precision-machined locating grooves, flanged bearings, and thrust ball bearings to achieve high-precision axial positioning. Closed-loop units are stacked layer by layer with thrust ball bearing supports, forming an interference-free kinematic chain. Silicone strips are attached to the envelope surfaces to enhance friction, while the servo-driven system enables wide-range angular adjustment. The DS5180SSG servo motor is employed, offering high torque output that fulfills the application requirements. The theoretical prototype shown in Figure 2 verifies structural feasibility, while the physical prototype (Figure 9) achieves both extension and flexion movements, exhibiting multi-layer enveloping surfaces with wide-range capability for grasping multi-size objects while maintaining structural compactness.

The motion accuracy of the mechanism is sensitive to tolerances and wear, primarily due to the error accumulation effect inherent in its serial-chain structure. Clearances in individual joints or dimensional errors in individual links propagate along the kinematic chain to the end-effector. In the design, the following strategies were implemented to mitigate this impact: (1) Precision Shafting Design: All revolute joints employ a composite support structure combining flanged bearings and thrust ball bearings. This design simultaneously withstands both radial and axial loads, achieving high-precision axial positioning and low-friction rotation, thereby fundamentally enhancing joint rigidity and motion accuracy. (2) Controlled Preload Assembly: In the shafting design of the revolute joints, the axial position of the shaft’s circlip groove is precisely set to control the fit clearance between the shaft and the bearing inner ring within a minimal range of 0.1–0.2 mm. Upon assembly, the elastic tension of the circlip generates a controlled axial preload. This aims to actively eliminate initial internal bearing play and, without inducing excessive frictional resistance, significantly suppresses shafting wobble caused by minor manufacturing errors, thus improving the positional stability of the end-effector in the multi-stage serial chain from the source.

**Figure 10:**
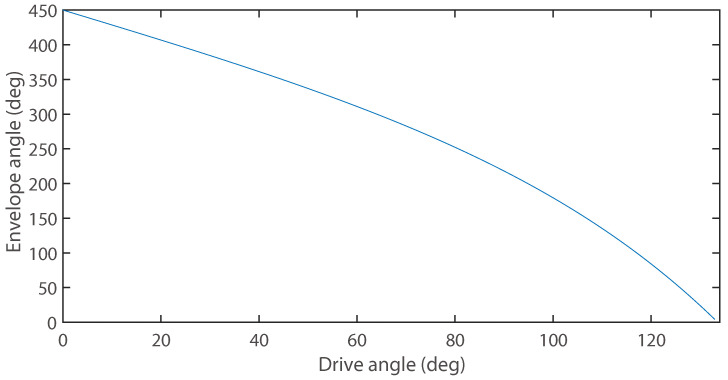
Envelope angle of one DOF N-layer reverse four-bar linkage (N = 6).

As shown in Figure 10, during the decrease of the driving angle $\theta_{1}$ from 133.6° to 0°, the mechanism gradually bends from the fully extended state to the bending limit, while the envelope angle increases from 0c to 450°. This behavior corresponds with Equation (15), exhibiting an approximately linear variation process. The linear fitting achieves an Adjusted R-square of 0.973, demonstrating high linearity.

The mechanism exhibits progressive enveloping characteristics at different driving angles $\theta_{1}$: At $\theta_{1}=133.6^{\circ }$, it extends into a straight configuration with slender profile for confined space operations; when $\theta_{1}$ decreases to $106.83^{\circ }$, it achieves $180^{\circ }$ envelopment satisfying the form closure condition; further reduction to $88.05^{\circ }$ enables $270^{\circ }$ envelopment; at $\theta_{1}=40.56^{\circ }$, it demonstrates hyper form closure characteristics with continuous closed-loop enveloping surfaces forming a decagonal contact profile and self-locking effect.

At the limit when $\theta_{1}$ approaches $0^{\circ }$ (as shown in Figure 8c), the mechanism reaches its theoretical maximum $450^{\circ }$ envelopment angle while entering a near-singular configuration at the critical transition between forward and reverse closed loops. For practical operation, we recommend maintaining $\theta_{1}\geq 20^{\circ }$ (corresponding to $406.8^{\circ }$ envelopment) to ensure 90.4% effective workspace utilization while avoiding control challenges from singular configurations.

## 4. Experimental Validation

**Figure 11:**
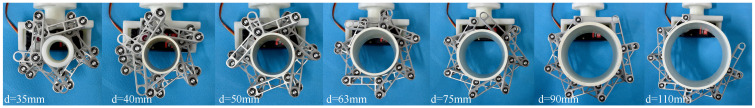
Experimental results for PVC hollow cylinders of different diameters.

**(1) Cylindrical Object Envelopment.** In the cylindrical grasping experiment, the designed prototype was placed on a table with target objects positioned within the finger’s enveloping range beforehand. The fingers gradually enveloped the objects through controlled motor rotation until successful grasping was achieved. Experimental results for PVC hollow cylinders of different diameters are shown in Figure 11.

The mechanism achieved stable grasping within a 35–110 mm diameter range: form-closure conditions were satisfied when grasping 75 mm, 90 mm and 110 mm cylinders; a self-locking loop structure formed when grasping 63 mm-diameter cylinders, demonstrating good anti-slip capability; full circumferential contact with the cylindrical surface was achieved through distributed multi-point contact forces establishing hyper form closure for 35–50 mm diameters.

**Figure 12:**
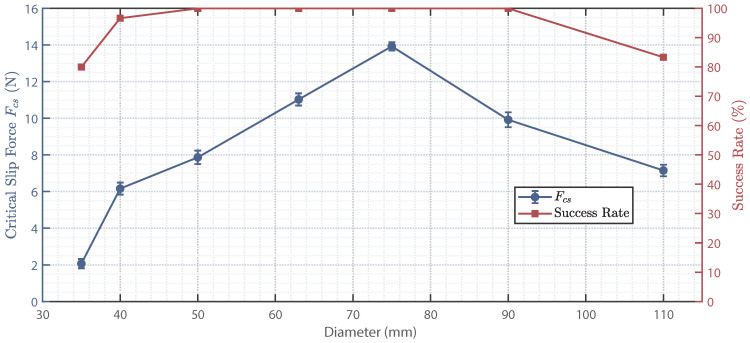
Grasp success rate and critical slip force for cylindrical objects of different diameters.

Furthermore, tests for both grasp success rate and critical slip force were conducted. A successful grasp was experimentally defined as follows: after the mechanism achieved full envelope and lift of a cylindrical object in a vertical posture, the grasped cylinder was tilted within $\pm 20^{\circ }$ and had to maintain this inclined state stably for 3 s without visible slip or detachment. Thirty grasp trials were performed for each object diameter. The critical slip force, which quantifies anti-slip performance, was identified as the first peak on the tension-displacement curve (obtained at a displacement rate of 2mm/s). Ten repeated measurements of the critical slip force were conducted along the cylindrical axis using a push-pull gauge. The corresponding grasp success rate and critical slip force for cylinders of different diameters are shown in Figure 12.

As shown in Figure 12, the grasp performance, characterized by the critical slip force ($F_{cs}$), does not vary monotonically with object diameter but exhibits a distinct peak at 75 mm. This reveals a key mechanical design insight: matching the target diameter optimizes the contact area and normal pressure distribution, maximizing the static friction potential under force-closure. In contrast, the grasp success rate remains at 100% across a broad diameter range (50–90 mm), declining only marginally at the smallest (35 mm) and largest (110 mm) diameters tested. Consequently, within the 50–90 mm range, the gripper achieves both high operational reliability and superior anti-slip capability, defining a clear optimization window for practical target object selection. This trend is attributable to the synergistic evolution of the contact area and normal pressure distribution with changing diameter.

**Figure 13:**
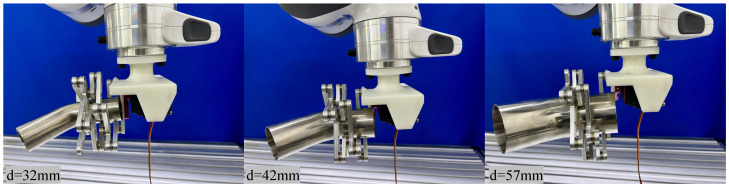
Experimental results for $45^{\circ }$ bent pipes (Ø32/42/57 mm).

**(2) Bent Pipe Grasping.** Subsequently, grasping experiments were conducted on 45° bent pipes with diameters of 32 mm, 42 mm, and 57 mm, as shown in Figure 13. The results verified the mechanism’s strong adaptability to tubular objects. By leveraging a self-locking-like mechanism to prevent slippage, the design fulfills the stable grasping requirements for standard pipe components in industrial pipeline installation and maintenance.

**Figure 14:**
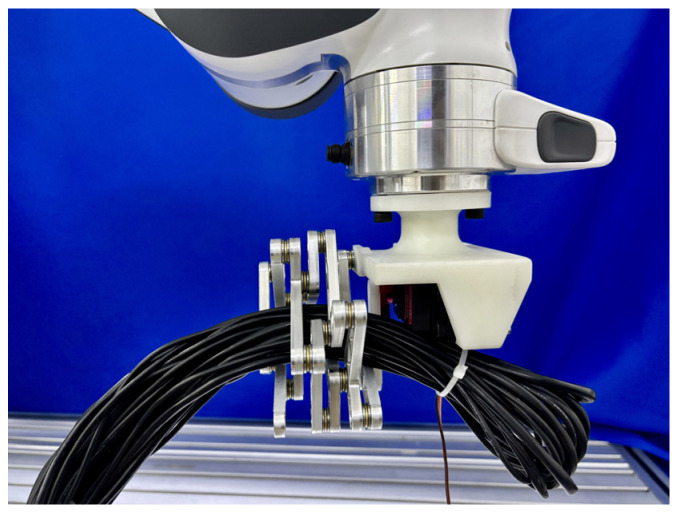
Experimental results for 35 mm-diameter rope.

**(3) Rope Manipulation.** In the final test, the mechanism demonstrated reliable grasping of a 35 mm-diameter rope bundle under a tensile load of 20 N applied along the rope’s axis, as shown in Figure 14. This capability confirms its applicability for industrial handling of flexible cables, hoses, and other non-rigid objects, providing a robust solution for scenarios such as aerospace cable routing and similar high-precision engineering applications.

The N-layer reverse four-bar linkage forms a closed-loop annular structure during object grasping, demonstrating distinct advantages over traditional two-finger gripping methods: its closed-loop geometric constraints enhance anti-release reliability by doubling the contact area to effectively enhance frictional forces, while enabling adaptive grasping of objects with diverse sizes and geometries. This mechanism provides a versatile and highly reliable grasping solution for industrial automation scenarios.

**(4) Prismatic Object Grasping.** The polygonal envelopment characteristic of the N-layer reverse four-bar linkage provides a unique advantage for grasping non-cylindrical objects. Unlike mechanisms that pursue a perfect circular envelope, our design generates an envelope composed of multiple discrete contact regions (conceptualized as polygon vertices). This characteristic enables it to naturally adapt to objects with edges or approximately polygonal cross-sections. The underlying principle is that the stability of hyper-form-closure does not rely on continuous, uniform circumferential contact, but on the formation of multiple non-collinear constraint points around the object, thereby satisfying the force-closure condition in the wrench space. For an object with a rectangular cross-section, the multiple links of the mechanism can sequentially conform to its adjacent surfaces, establishing a stable force lock at the edges. Crucially, the hyper-form-closure remains effective even with non-uniformly distributed contact points, provided these discrete points constrain all potential motions (both translational and rotational) of the object.

**Figure 15:**
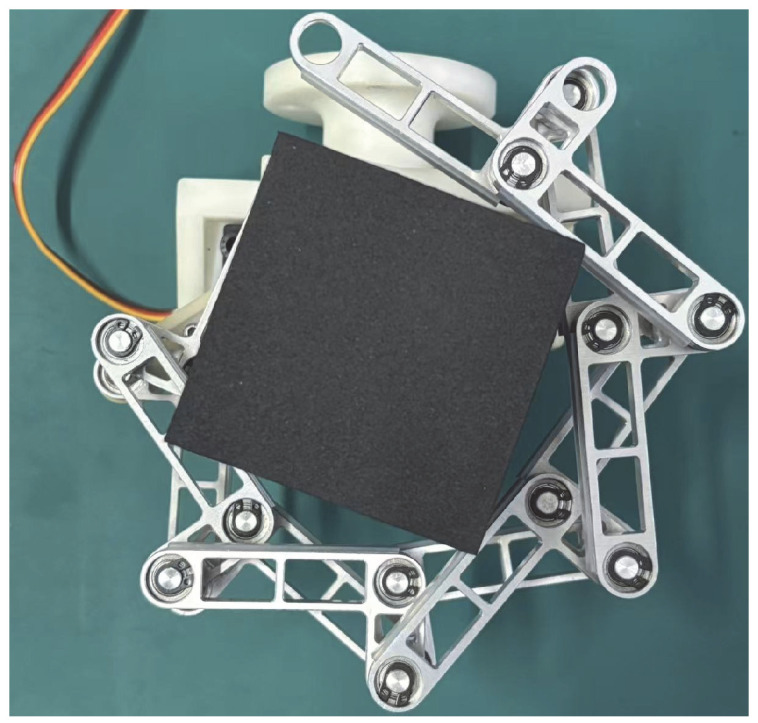
Experimental results for a square prism (60 mm × 60 mm).

To evaluate the gripper’s capability and the robustness of hyper-form-closure for non-cylindrical objects, grasping experiments were performed on a rectangular prism. Figure 15 shows that the mechanism successfully grasped a prism with a square cross-section (60 mm × 60 mm). During grasping, utilizing its intrinsic polygonal envelopment characteristic, links at different layers formed effective contacts with distinct faces or edges of the prism, resulting in stable hyper-form-closure grasping. This result confirms the effectiveness and robustness of the grasping strategy based on discrete contact points for handling non-cylindrical objects.

**(5) Load-Bearing Capacity.** To intuitively verify the load-bearing capacity of the gripper, a stepped load-bearing experiment was conducted. After grasping the object, the envelope axis of the gripper-load assembly was oriented horizontally (i.e., perpendicular to the gravity vector), establishing a suspended loading condition. Gravitational loads of 5 N, 10 N, and 15 N were subsequently applied to the object. The results demonstrate that the gripper could successfully grasp and stably lift the object under all tested loads without any slip or instability. This confirms that the gripper maintains fully reliable performance within a load range of at least 15 N, providing direct experimental justification for its load rating in practical applications.

## 5. Discussion

### 5.1. Comparative Analysis with Existing Actuators

The performance comparison between the N-layer reverse four-bar linkage proposed in this paper and existing actuators is shown in Table 1. The 6-bar finger designed by the authors [22] exhibits strong gripping force but suffers from a limited maximum envelope angle (213.47°) and narrow enveloping range (25–60 mm), making it unsuitable for large targets. The pneumatic soft spiral gripper [23] achieves 540° wrapping (1.5 turns), yet its silicone precision casting process complicates manufacturing and delivers only moderate gripping force. The bio-inspired spring origami structure [24] displays spring-like behavior at rest, locking into a hook configuration upon compression. While its envelope angle exceeds 360°, the gripping force remains insufficient. The seahorse-inspired cable-driven linkage robot [25] requires coordinated control of four cables, presents moderate manufacturing complexity, and achieves 517.6° wrapping (approximately 1.44 turns), but its 60–100 mm diameter range and control complexity hinder industrial adoption.

**Table 1:** Comparative Results with Existing Actuators.

Name	Num of DOF	Maximum Envelope Angle	Envelope Cylindrical Diameter Range	Simple Structure
6-bar finger designed by the author [22]	1	213.47°	25–60 mm	↗
Pneumatic soft spiral gripper [23]	1	0–540°	1–8 mm	↘
Bio-inspired spring origami structure [24]	1	>360°	about 38 mm	↘
Seahorse-inspired cable-driven linkage robot [25]	4	517.6°	60–100 mm	⟶
N-reverse four-bar linkage (N = 6) in Figure 9	1	$\left(N-1\right)\cdot \alpha_{1}$	35–110 mm	↗

↗: Advantage; ⟶: Moderate; ↘: Inferiority.

In contrast, our mechanism utilizes single-degree-of-freedom actuation to switch between extension, bending, and wrapping modes. Its envelope angle is parametrically controlled by $\left(N-1\right)\cdot \alpha_{1}$ (reaching 450° at $\alpha_{1}=90^{\circ }$, N=6 and 540° at N=7). All links employ identical standardized components, significantly reducing manufacturing costs and assembly complexity. With a broad enveloping range of 35–110 mm covering industrial piping standards, it provides a highly adaptable solution for pipeline assembly and equipment maintenance scenarios.

### 5.2. Non-Uniform Design and Its Feasibility

This study employs a uniform self-similar topology primarily due to its simplicity in modeling, manufacturing, and control, which facilitates proof of principle. However, adopting a tapered non-uniform design (e.g., gradually shortening link lengths layer by layer) holds significant potential: it can enhance adaptability to objects of varying sizes (with distal links conforming closely to smaller objects and proximal links bearing the main load) and improve suitability for confined spaces. Although such a design introduces complexities in modeling and manufacturing, it provides an important direction for improving next-generation twining mechanisms.

**Figure 16:**
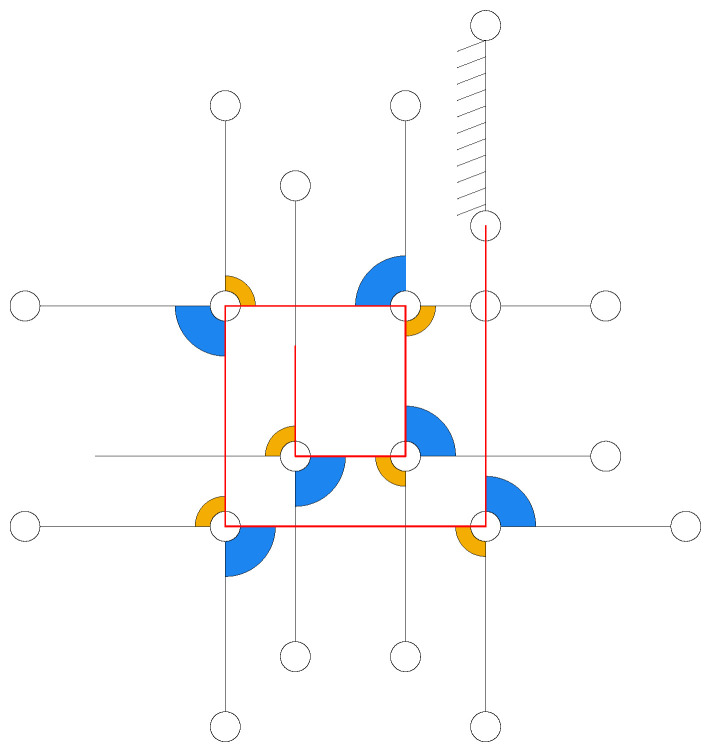
Non-uniform design of the N-layer reverse four-bar linkage (N=6, $\alpha =90^{\circ }$, short links: 20 mm, long links: tapering from 50 mm by 4 mm per layer). The red line indicates the envelope curve at the bending limit posture.

We conducted simulations of an N-layer reverse four-bar linkage with non-uniform design based on previous parameters. The number of closed loops *N* is set to 6, all α angles are $90^{\circ }$, the short links $l_{1}$ and $l_{3}$ are fixed at 20 mm, and the long links $l_{2}$ and $l_{4}$ in the first loop are set to 50 mm, decreasing by 4 mm with each subsequent layer. This non-uniform configuration can still achieve full extension and bending motions. Its bending limit state is shown in Figure 16.

As can be seen from Figure 16, the envelope curve of the bending limit posture exhibits a spirally tightening shape. Since $\alpha =90^{\circ }$, this envelope curve manifests as a square spiral. Its geometric generation can be described as follows: starting from an initial segment length $L_{0}=50$ mm, each subsequent segment is obtained by a $90^{\circ }$ clockwise rotation and a length reduction of 4 mm, repeated N−1 times. By progressively decreasing the link lengths layer by layer, this configuration achieves a gradual inward tightening of the envelope curve, thereby enhancing its conformance capability to targets of different diameters while maintaining structural stiffness.

In future work, we will explore feasible pathways toward achieving “structural non-uniformity with functional adaptability” by combining parametric optimization and novel materials.

### 5.3. Cable-Driven N-Layer Reverse Four-Bar Linkage

By overlapping link $B_{n+1}C_{n}$ of closed-loop *n* with link $A_{n+1}B_{n+1}$ of the subsequent closed-loop in Figure 4, a special N-reverse four-bar closed-loop mechanism is constructed. Each closed-loop in this mechanism possesses an independent degree of freedom, resulting in a total of N degrees of freedom for the overall system. Short links are extended outward with openings on both sides, adopting a bilateral cable-driven differential control scheme that enables directional bending through coordinated motion of two 42-stepper motors. Cables are wound from inside to outside, with mechanical design ensuring opposite winding directions on both sides: during leftward curling, both motors rotate counterclockwise (left reeling in, right paying out); during rightward curling, both rotate clockwise (right reeling in, left paying out), achieving winding motion through simple synchronized control.

**Figure 17:**
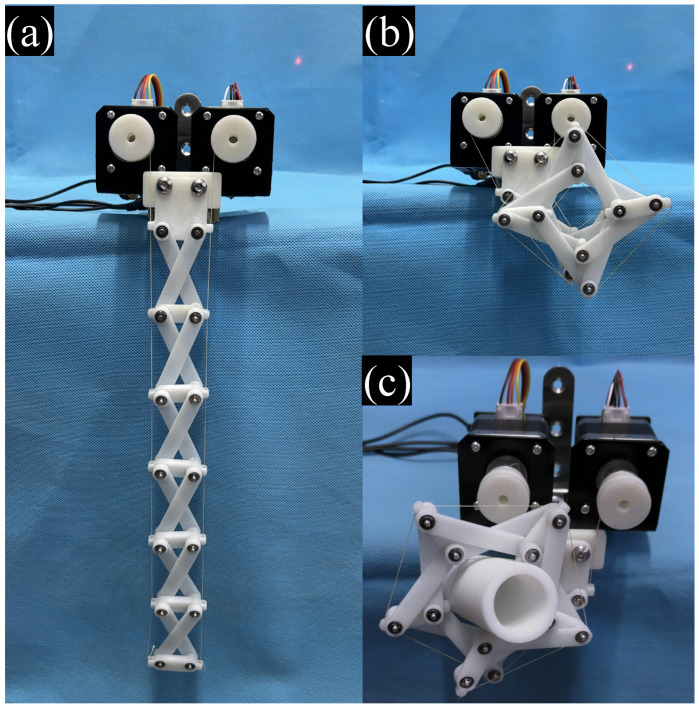
Cable-Driven N-Layer Reverse Four-Bar Linkage Prototype: (**a**) Extended State, (**b**) Rightward Curling, (**c**) Enveloping a Cylindrical Object.

Structurally, the frame, links, and cable reels are manufactured via 3D printing, with reels connected to transmission shafts through flanges. As shown in Figure 17a,b, this configuration enables switching between extended and curled states. Figure 17c demonstrates its effectiveness in grasping cylindrical objects, verifying the mechanism’s excellent enveloping grasping capability. This design combines differential cable drive with a compact bearing structure, ensuring motion accuracy while achieving low-cost manufacturing, providing a reliable solution for industrial grasping applications.

It is important to note that the cable-driven prototype exhibits significantly lower sensitivity to joint clearance compared to a rigid, directly driven configuration. In the cable-driven system, as long as the motors precisely control the cable length and tension, the uncertainty introduced by minor joint clearances can be absorbed by the flexibility of the cables and does not decisively affect the macroscopic motion trajectory of the end-effector.

## 6. Conclusions

This paper establishes the kinematic general formula and enveloping angle relationship for N-layer Reverse Four-Bar Linkage, analyzing the influence of structural parameters on configurations. The mechanism maintains excellent kinematic stability during multi-layer expansion by utilizing self-similar topological characteristics. Prototype experiments validate the design’s application potential in various scenarios, particularly its grasping capability for multi-sized cylindrical objects. Compared with traditional two-finger grippers, the closed-loop design significantly improves anti-slip reliability through geometric constraints while enhanced contact area ensures grasping stability. The stepped arrangement effectively solves interference problems in multi-link mechanisms. Future work will focus on vision-based intelligent grasping control strategies to further enhance the mechanism’s practical value in flexible manufacturing and logistics sorting applications.

## Data Availability

The data that support the findings of this study are available from the corresponding author upon reasonable request.
